# Early competition shapes maize whole-plant development in mixed stands

**DOI:** 10.1093/jxb/ert408

**Published:** 2013-12-04

**Authors:** Junqi Zhu, Jan Vos, Wopke van der Werf, Peter E. L. van der Putten, Jochem B. Evers

**Affiliations:** Centre for Crop Systems Analysis, Wageningen University, PO Box 430, 6700 AK, Wageningen, The Netherlands

**Keywords:** Coordination of development, leaf development, phyllochron, plastochron, shade avoidance, wheat–maize intercropping.

## Abstract

Mixed cropping is practised widely in developing countries and is gaining increasing interest for sustainable agriculture in developed countries. Plants in intercrops grow differently from plants in single crops, due to interspecific plant interactions, but adaptive plant morphological responses to competition in mixed stands have not been studied in detail. Here the maize (*Zea mays*) response to mixed cultivation with wheat (*Triticum aestivum*) is described. Evidence is provided that early responses of maize to the modified light environment in mixed stands propagate throughout maize development, resulting in different phenotypes compared with pure stands. Photosynthetically active radiation (PAR), red:far-red ratio (R:FR), leaf development, and final organ sizes of maize grown in three cultivation systems were compared: pure maize, an intercrop with a small distance (25cm) between maize and wheat plants, and an intercop with a large distance (44cm) between the maize and the wheat. Compared with maize in pure stands, maize in the mixed stands had lower leaf and collar appearance rates, increased blade and sheath lengths at low ranks and smaller sizes at high ranks, increased blade elongation duration, and decreased R:FR and PAR at the plant base during early development. Effects were strongest in the treatment with a short distance between wheat and maize strips. The data suggest a feedback between leaf initiation and leaf emergence at the plant level and coordination between blade and sheath growth at the phytomer level. A conceptual model, based on coordination rules, is proposed to explain the development of the maize plant in pure and mixed stands.

## Introduction

Intercropping is widespread in large parts of China, Africa, and Latin America (Vandermeer, 1989, 2011). It has important advantages compared with single crop systems: greater crop production per unit land (overyielding) (Li *et al.*, 2007), potential for improved water and nutrient capture (Morris and Garrity, 1993*a*, b), enhanced pest and disease suppression (Zhu *et al.*, 2000), and overall lower production risks (Rao and Singh, 1990). Adaptations in plant architecture and physiology are likely to contribute to the often reported overyielding (Lithourgidis *et al.*, 2011), but these adaptations have not been analysed (Connolly *et al.*, 2001). There is increasing interest in mixed cultivation systems in developed countries, to strengthen the ecological basis of agriculture and exploit the advantages of intercropping and agroforestry (Eichhorn *et al.*, 2006; Lichtfouse *et al.*, 2009; Pelzer *et al.*, 2012).

Light competition may be severe in mixed stands. Plants need to adapt to either tolerate (Gommers *et al.*, 2013) or avoid shading by neighbours (Franklin and Whitelam, 2007). For plants showing shade avoidance, alterations in both light quality and light quantity can invoke a suite of responses, including enhanced stem and petiole elongation, reduced branching, and more erect leaf angles (Sultan, 2010; de Wit *et al.*, 2012). Little is known about the consequences of local adaptation (e.g. enhanced sheath length) on later development and how this shapes and influences the development of whole-plant architecture. Understanding the development of whole-plant architecture (e.g. leaf appearance rate and final organ sizes) in mixed plant systems is of great importance for analysing whole-plant fitness and productivity of a component species in a mixed system and of the system as a whole.

A plant is built by the repeated formation, expansion, and (partial) senescence of phytomers (Forster *et al.*, 2007). Growth responses of whole plants are realized by changes in the growth at phytomer level (Beemster *et al.*, 2003) with control at the plant level via hormones and sugar levels (de Kroon *et al.*, 2005, 2009). A phytomer of maize (*Zea mays*) consists of an internode with an axillary bud at the bottom, and a node, a leaf sheath, and blade at the top. New phytomers are created at the shoot apex. Each component of the phytomer unit differentiates, grows, appears, and senesces with coordination among the components (McMaster, 2005).

During the vegetative stages of maize and rice (*Oryza* spp.), blade tip emergence (defined as the blade tip growing past the highest collar; the collar marking the border between the sheath and blade) is associated with the initiation of the associated sheath (defined as the moment when the sheath length passes 1mm) (Andrieu *et al.*, 2006; Parent *et al.*, 2009). In maize, collar emergence of a leaf, defined as its collar growing past the collar of the preceding leaf, is associated with a decline in elongation rate of the sheath and an increase in elongation rate of the internode, with the sum of the two remaining the same (Fournier and Andrieu, 2000*a*, *b*). It has been shown in grasses that blade and sheath length of a leaf are positively associated with the length of the whorl of mature sheaths through which the leaf grows (Davies *et al.*, 1983; Wilson and Laidlaw, 1985; Skinner and Nelson, 1994; Casey *et al.*, 1999). The length of the whorl affects several attributes of a leaf, such as final length, elongation rate, and length of the growing zone defined as the part in which cells divide and elongate (Durand *et al.*, 1995; Fournier *et al.*, 2005).

Leaf emergence rate in grasses is determined by the rate of leaf initiation at the apex, leaf elongation rate, and the whorl length of mature sheaths of previous phytomers through which this leaf emerges (Skinner and Nelson, 1995). In maize, high population density and a low red:far-red ratio (R:FR) decelerate leaf emergence and increase sheath growth (Andrieu *et al.*, 2006; Page *et al.*, 2011), whereas leaf emergence rate increases with the daily sum of incident photosynthetically active radiation (PAR) (Birch *et al.*, 1998c; Padilla and Otegui, 2005). Conservative relationships between leaf emergence and leaf initiation have been found across hybrids and environments in wheat (*Triticum aestivum*) (Kirby, 1990), rice (Nemoto *et al.*, 1995), sunflower (*Helianthus annuus*) (Sadras and Villalobos, 1993), and maize (Kiniry *et al.*, 1983; Padilla and Otegui, 2005). The conservative relationship between the numbers of emerged and initiated leaves suggests coordination between the rates of leaf emergence, leaf growth, and leaf initiation at the plant level.

All of these adaptive responses may be involved in the response of plants to mixed cropping, but, as yet, ecophysiological research on intercrop performance has not considered the possibility of an effect of mixed cropping on the regulation of plant development. It is expected that plants in intercrops develop different structures in response to the changed light environment, while structural responses of different phytomers on the same plant are coordinated. What are these structural adaptations, and how are they coordinated between different phytomers on the same plant?

To answer these questions, a detailed analysis was conducted of maize development in three contrasting cultivation systems (henceforth: treatments): pure maize, an intercrop with a small distance (25cm) between wheat and maize plants, and an intercrop with a large distance (44cm) between the wheat and the maize. The structural development of the maize plants in the three systems was characterized by measuring leaf appearance, rate and duration of leaf elongation, collar emergence, and final sizes of the blade and sheath of each phytomer. Generic coordination rules were inferred from the data.

## Materials and methods

### Experiment set-up

**Fig. 1.**
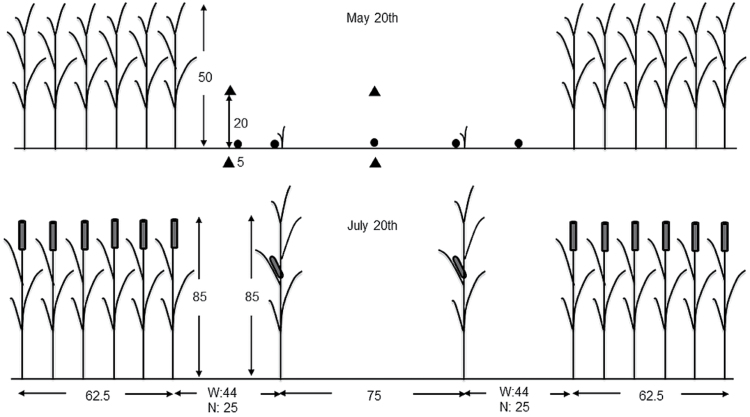
Cross-row profile of a wheat–maize intercrop (unit: cm). Wheat was grown in 62.5cm wide strips consisting of six rows at a distance of 12.5cm. Maize was grown in strips of two rows, with 75cm between the rows. The distance between maize and wheat was 44cm (wide intercrop: W) or 25cm (narrow intercrop: N), resulting in contrasting levels of interaction between wheat and maize. Wheat was sown on 9 March, and harvested on 10 August, while maize was sown on 11 May, and harvested on 14 October. On 20 July (675 °Cd), maize in wide intercrop reached the same height as the wheat. Dots indicate placement of PAR sensors; triangles represent placement of thermocouples.

All measurements were made under ambient conditions in a field experiment in Wageningen, The Netherlands (51°59′20′′N, 5°39′16′′E) from March to October in 2011. Maize and wheat were grown in single and mixed stands on a sandy soil [N supply capacity, 96kg N ha^–1^ year^–1^; organic matter, 5.9% with a C/N ratio of 15; soil mineral N content before sowing (0–60cm), 28kg ha^–1^]. Maize growth in three treatments was compared: (i) monoculture maize at a row distance of 75cm and a population density of 9.87 plants m^–2^; (iii) wide intercrop with 44cm distance between adjacent wheat and maize rows; and (iii) narrow intercrop with 25cm between wheat and maize rows (Fig. 1). Intercropped maize was grown in strips of two rows, while intercropped wheat was grown in strips of six rows (Supplementary Fig. S1 available at *JXB* online). Row distance was 12.5cm in wheat and 75cm in maize. Intercrop plots included two maize strips, three wheat strips, and a maize border at each side. Plot size was 6×6 m for both monoculture and wide intercrop. For narrow intercrop, the plot width was 4.9 m (Supplementary Fig. S1). Row direction was north–south. Each treatment was replicated three times.

Wheat ‘Tybalt’ was sown on 9 March 2011, and harvested on 10 August 2011. Maize ‘LG30208’ was sown on 11 May and harvested on 14 October. Fertilizer was applied homogeneously throughout the experiment. The first application was done on 5 April, after wheat emergence: 15kg P ha^–1^, 37kg K ha^–1^, 14kg Mg ha^–1^, and 46kg N ha^–1^. Furthermore, 75kg N ha^–1^ was top-dressed on 20 May shortly after maize emergence. An additional 50kg N ha^–1^ was top-dressed on 10 June. Weeds were controlled mechanically after wheat emergence and chemically by ‘primstar’ and ‘MCPA 500’ on 27 April and by ‘biathlon’ and ‘kart’ on 3 June. Fungicide ‘prosaro’ and insecticide ‘decis’ were applied on 10 June in both wheat and maize.

### Temperature measurements and calculation of thermal time

Temperature was recorded (Datataker DT600, Data taker Data Loggers, Cambridgeshire, UK) with shielded thermocouples (type T, TempControl Industrial Electronic Products, Voorburg, The Netherlands) at 10min intervals. Thermocouples were placed at 5cm depth, and at 20cm above the soil surface within the canopy. Four thermocouples, two in the canopy and two in the soil, were placed at two locations in the maize strip, between the rows of maize and between the adjacent maize and wheat rows, in both intercrop treatments (triangles in Fig. 1). In monoculture maize, two thermocouples were placed between the rows, one in the canopy and one in the soil. Only slight differences in temperature were found between treatments and positions. Thermal time (°Cd, degree days) was calculated on an hourly basis from sowing, considering a base temperature for maize development of 8 °C (Birch *et al.*, 1998*a*). Averaged soil thermal time for different positions from sowing to 1 July was 8 °Cd less in wide intercrop than in monoculture and 28 °Cd less in narrow intercrop than in monoculture. Average soil temperature over positions and treatments was used for temperature accumulation before jointing (1 July), when the apex was still below the soil surface, while average canopy temperature over positions and treatments was used for temperature accumulation after jointing.

### Plant selection

In each plot, 12 similar maize plants were tagged when blade 2 was visible. Four of these were used for non-destructive observations. The remaining eight were used in two destructive samples, and their location was chosen such that sampling effects on plants used for non-destructive observations were minimized. To compare plants of similar developmental pattern, 49 maize plants with the predominant final leaf number in each treatment were selected from those initially tagged (see the Results for details) to analyse blade elongation and final organ size.

### Blade dynamics

Leaves were counted acropetally starting from the bottom leaf. The number of visible, mature, and dead leaves as well as the exposed length of all immature leaf blades were measured twice per week. Tip appearance and collar emergence were considered to occur midway between the last observation when the event had not yet occurred and the first observation date on which the event had occurred. Emergence was defined as the moment a blade tip or collar had grown past the highest collar of the preceding sheaths. Tip appearance was defined as the moment the blade tip had visibly appeared out of the whorl formed by preceding growing blades, when looking at a horizontal angle into the whorl. A blade was considered mature when its collar had emerged from the sheath tube formed by preceding phytomers. Phyllochron (i.e. the thermal time between appearances of successive leaf blades) was estimated as the slope of the linear relationship between thermal time at tip appearance and phytomer rank (°Cd leaf^–1^). Regressions were made using the linear mixed-effects model (lme) in the ‘nlme’ package of the R programming language (R Core Team, 2012) with plot and plant (nested in plot) as random effects. The same method was used for analysing the relationship between final blade length and length of the encapsulating sheath.

### Blade elongation duration and elongation rate

Blade dynamics data were further used for estimating the duration of blade elongation and to calculate the average rate of elongation. Blade elongation duration was estimated by fitting the beta function (Equation 1) (Yin *et al.*, 2003).


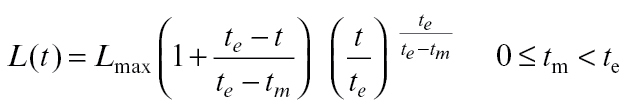
(1)

where *L*(*t*) is the measured blade length at thermal time *t* (°Cd) and *L*_max_ is the final blade length (cm). *t*_e_ is the time when the final blade length was reached (°Cd) corresponding to the elongation duration from tip appearance to collar emergence in the measurement; *t*_m_ is the time when growth rate peaks (°Cd). Parameters *t*_m_ and *t*_e_ were estimated (Supplementary Fig. S2 at *JXB* online) for each single blade using non-linear curve fitting with least squares (‘lsqnonlin’) in MATLAB 2012a (The MathWorks Inc., Natick, MA, USA). The average elongation rate was calculated as the ratio of final blade length and estimated elongation duration (*t*_e_).

### Final organ size

The sizes of all fully grown organs (blade, sheath, and internode) of each maize phytomer were measured destructively on two sampling occasions. Blade width was measured at the widest cross-section. The first sampling (656 °Cd) was at collar emergence of leaf 10 in monoculture maize; the second sampling (1261 °Cd) was after maturity of the final leaf. Final blade length of phytomer ranks 1 and 2 and final sheath length of rank 1 were not recorded due to their advanced senescence at the time of the first sampling.

### Red:far-red ratio (R:FR)

Weekly measurements were made of the R:FR at ~2cm above soil level around noon, using the Skye SKR100/116 Fibre Optic Probe Measuring System (Skye Instruments Ltd, Powys, UK). The device was equipped with a glass fibre probe that measured R:FR at its tip, with an angle of view of 40 ° relative to the soil surface. Measurements were made parallel to the soil surface with the sensor backing against the plant and facing north, east, south, and west. The average of the four values was used for analysis.

A four-parameter logistic function (Equation 2) was used to fit the data on R:FR versus thermal time using the ‘nlinfit’ function of MATLAB.


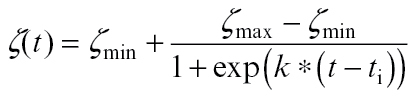
(2)

where ζ(*t*) is the measured R:FR at thermal time *t*. ζ_min_ and ζ_max_ are the lower and upper asymptotes (dimensionless), *k* is the slope at the inflection point (°Cd^–1^), *t* is thermal time (°Cd), and *t*_i_ is the thermal time of the inflection point (°Cd).

### Photosynthetically active radiation (PAR)

Light penetration at 2cm above soil level was measured once per week around noon with a 1 m long light-sensitive bar that was held parallel to the crop rows (SunScan Canopy Analysis System; Delta T Devices, Cambridge, UK). A reference PAR sensor was placed just above the canopy. Four fixed positions in each plot were measured in monoculture (two replicates in the middle of rows, and two directly adjacent to the plants with the row) and five in each of the intercrop treatments (dots in Fig. 1). A weighted mean fraction of the PAR value of the four or five positions was used in further analysis. The weighting factors of different positions in the intercrop plots were calculated by their representative length; see Supplementary Method S1 at *JXB* online for details.

A four-parameter logistic function (Equation 3) was used to model the fraction of incoming PAR reaching soil level as a function of thermal time:


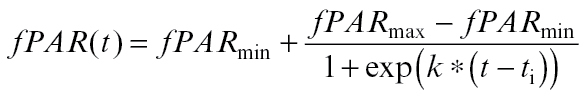
(3)

where *fPAR*(*t*) is the fraction of the PAR at soil level at thermal time *t*. *fPAR*_min_ and *fPAR*_max_ are the lower and upper asymptotes (dimensionless), *k* is the slope at the inflection point (°Cd^–1^), *t* is thermal time (°Cd), and *t*_i_ is the thermal time of the inflection point (°Cd). The function was fitted to data using the ‘nlinfit’ function of MATLAB.

### Tassel initiation and silking time

Tassel initiation time (i.e. the switch from the vegetative to the generative phase) is estimated as the time when the final leaf was initiated at the apex. The timing of this switch cannot be observed macroscopically without dissection, and was therefore calculated from a linear regression between leaf initiation and leaf appearance (Supplementary Table S2 at *JXB* online) (Padilla and Otegui, 2005). Silking time was defined as the time at which 75% of plants have silks visible (Hanway, 1963).

### Comparison of leaf initiation rate and leaf appearance rate between treatments

Average leaf initiation rate (LIR) can be estimated by dividing the total number of initiated leaves by the thermal time from germination to tassel initiation. In order to assess the stability of the relationship between LIR of the monoculture and intercrop treatments, the ratio between LIR in monoculture and LIR in wide and narrow intercropping was calculated. As the thermal time to tassel initiation was similar among treatments, this ratio between LIR values could be estimated by simply taking the ratios of the number of initiated leaves (Equation 4):


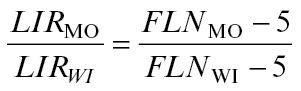
(4)

where *LIR* is leaf initiation rate in monoculture (MO), wide intercropping (WI), or narrow intercropping (NI; not shown here). *FLN*_MO_, *FLN*_WI_, and *FLN*_NI_ represent the final leaf numbers in the three different treatments. It is assumed that five leaf initials are present in the embryo (Padilla and Otegui, 2005).

The leaf appearance rate (LAR) is the reciprocal of phyllochron. The ratio between LAR in monoculture and LAR in wide and narrow intercropping was calculated by taking the ratios of the reciprocal of the phyllochron in each treatment.

### Statistics

The data were analysed with linear mixed models to account for random effects and nesting in the data. The linear mixed model also takes into account the fact that there were slightly different numbers of plants per plot, due to the random nature of the plant selection used. Two types of linear mixed models were used in the analysis, depending on the data structure. In the first type of analysis, there was only one measurement per plant. In this case, the plant was the unit of analysis, and the data were analysed with treatment as fixed effects and block and plot as random effects. This type of model was used for analysing final organ sizes, and blade elongation durations and rates. In the second type of analysis, there were multiple measurements per plant included in the analysis. In this case, the data were analysed with phytomer rank and treatment as fixed effects, and block, plot, and plant as hierarchically nested random effects. This applies to the regression of phyllochron data. As none of the analyses with linear mixed models yielded a significant block effect, this effect was dropped from all models. The mixed effects model with plot and plant as random effects was used for analysing all data with multiple measurements per plant included in the analysis, and a model with only plot as random effect was used to analyse data with single measurement per plant. Multiple comparisons of treatments for final organ sizes, and blade elongation durations and rates were done by means of least significant differences (LSD test, *P*=0.05) in the ‘agricolae’ package of R, after the treatment effects had been found significant using the mixed effects linear model. The mean square error and associated degrees of freedom required by the LSD function of R were obtained from the generalized least squares (gls) function with the restricted maximum likelihood (REML) method in the ‘nlme’ package in R. The experiment-wise rate of rejecting null hypotheses increases above the specified level α when multiple comparisons are made. Use of LSD values here is justified due to the low number of treatments, and thus comparisons, but caution should nonetheless be used when interpreting marginally significant results.

## Results

### Phenology

Maize emerged ~60 °Cd after sowing, at which time the wheat was ~50cm high. Maize in the wide intercrop treatment started to overtake wheat in height at 675 °Cd (Fig. 1). At this time, the height of maize (measured from the soil surface up to the highest point at which the whorl of growing leaves still forms a completely closed tube) was 125cm in monoculture, 85cm in wide intercrop, and 70cm in narrow intercrop. Maize in the narrow intercrop overtook wheat in height at 735 °Cd. The estimated tassel initiation time was 283 °Cd in monoculture, 295 °Cd in wide intercrop, and 310 °Cd in narrow intercrop. Observed silking time was 780 °Cd in monoculture, 830 °Cd in wide intercrop, and 864 °Cd in narrow intercrop.

Average final leaf number was 15.3±0.08 in monoculture, 14.0±0.26 in wide intercrop, and 13.6±0.35 in narrow intercrop (Table 1). The most common number of leaves was 15 in monoculture, 14 in wide intercrop, and 13 or 14 in narrow intercrop (Table 1). For subsequent analyses on the characteristics of phytomers of individual plants, a subsample of plants with 15 leaves for monoculture, plants with 14 leaves in wide intercrop, and an equal number of plants with 13 and 14 leaves in narrow intercrop were used, representing the modal leaf numbers in the three treatments. For the selected plants, the position of the subtending leaf of the cob was at rank 10 for monoculture, and rank 9 for both wide intercrop and narrow intercrop. The ratio between leaf initiation rates in monoculture and intercropping was 1.11 for wide intercropping and 1.25 for narrow intercropping.

**Table 1.** Final leaf number distribution of three treatments in non-destructive observation (n=12), destructive samples (n=12), and random count in the field (n=40 for monoculture, n=30 for both wide and narrow intercrop treatments)

Sample	Treatment	Number of leaves				
		12	13	14	15	16
Non-destructive samples (*n*=12)	Monoculture				8 (67%)	4 (33%)
	Wide intercrop			9 (75%)	3 (25%)	
	Narrow intercrop	1(8%)	7 (58%)	4 (33%)		
Destructive samples (*n*=12)	Monoculture				11 (92%)	1 (8%)
	Wide intercrop		1 (8%)	7 (58%)	4 (33%)	
	Narrow intercrop		3 (25%)	7 (58%)	2 (17%)	
Field random count (*n*=40 or 30)	Monoculture				24 (60%)	9 (22%)
	Wide intercrop		7 (23%)	15 (50%)	8 (27%)	
	Narrow intercrop	3 (10%)	10 (33%)	12 (40%)	5 (17%)	

### Leaf appearance and maturity

**Fig. 2.**
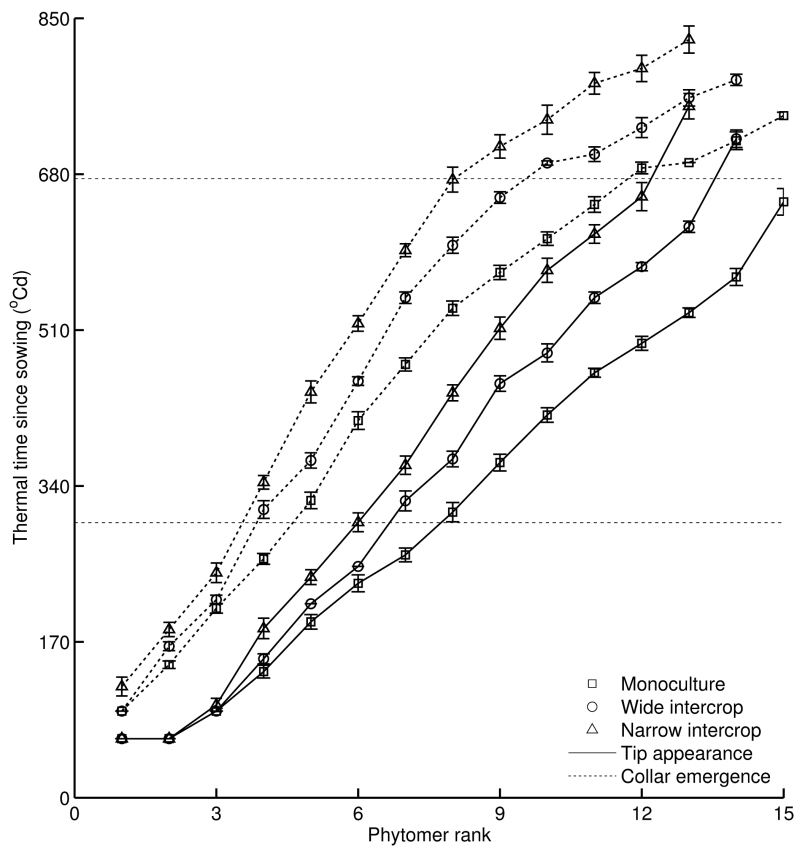
Moment of blade tip appearance (solid lines) and collar emergence (dotted lines) of maize in monoculture (squares, *n*=8), wide intercrop (circles, *n*=9), and narrow intercrop (triangles, *n*=7) versus phytomer rank. The upper dotted line (*y*=675 °Cd) indicates the time when maize in the wide intercrop became taller than wheat. The lower dotted line (*y*=300 °Cd) indicates tassel initiation time. Error bars indicate the SE.

From phytomer 4 onwards, blade tip appearance diverged among treatments (Fig. 2, solid lines). Average phyllochron from rank 2 onwards was 44.2±0.5 °Cd in monoculture, 54.2±0.7 °Cd in wide intercrop, and 62.8±0.8 °Cd in narrow intercrop. The ratio between leaf appearance rates in monoculture and intercropping was 1.23 for wide intercropping and 1.42 for narrow intercropping. At the time of tip appearance of rank 14 in monoculture, plants in wide intercrop had ~12 leaves and plants in narrow intercrop had ~10 leaves (Fig. 2).

Divergence of collar emergence occurred across treatments at low ranks (up to rank 8) (Fig. 2, dotted lines). The slope for ranks 3–8 was 67.4±1.3 °Cd in monoculture, 77.5±1.7 °Cd in wide intercrop, and 85.1±1.6 °Cd in narrow intercrop. For ranks beyond rank 8, collars emerged at similar thermal time intervals in the three treatments (27.6±1.1 °Cd in monoculture, 25.3±1.4 °Cd in wide intercrop, and 28.9±2.0 °Cd in narrow intercrop).

### Final size of organs

**Fig. 3.**
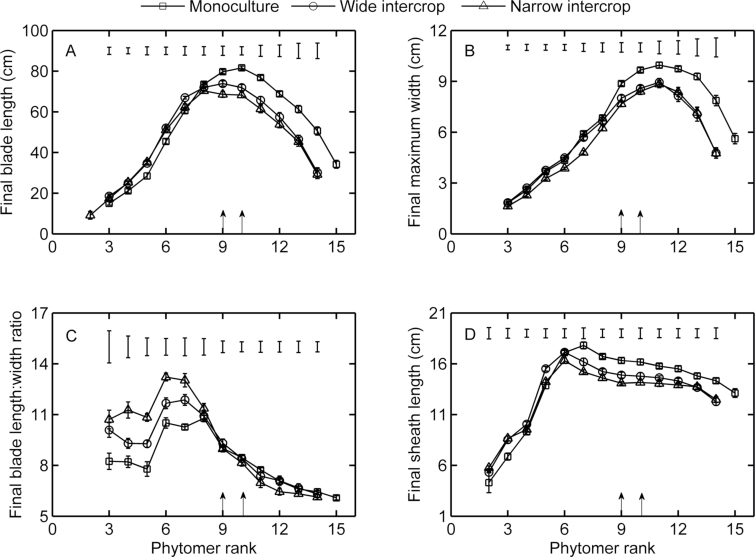
(A) Final blade length, (B) final blade width, (C) final blade length:width ratio, and (D) final sheath length in monoculture (squares, *n*=11), wide intercrop (circles, *n*=7), and narrow intercrop (triangles, *n*=7) versus phytomer rank. Error bars indicate the SE. Top bars represent the LSD (*P*=0.05). Arrows represent the rank of the subtending leaf of the cob at two intercrops (left arrow) and at monoculture (right arrow) in each panel.

Monoculture plants had the shortest blades in ranks up to 7 (Fig. 3A), but the longest blades in ranks beyond 8. Differences between treatments were significant for all ranks except 2 and 8 (LSD test at significance level of 0.05). However, for upper ranks, this effect was confounded with differences in final leaf number between the treatments. Narrow intercrop plants had the smallest blade width for ranks up to 7. For ranks beyond 7, monoculture plants showed a significantly larger final blade width than the other treatments (Fig. 3B, significant for all ranks except rank 8). Leaf shape, represented by the ratio between final blade length and width, showed significant differences between treatments for ranks up to 7 (Fig. 3C). In contrast, for ranks beyond 7, a similar ratio was found across treatments, even though plants in monoculture and intercrops differed in their final dimensions. Across all treatments, monoculture plants had the smallest final sheath lengths in ranks up to 5 (Fig. 3D). Monoculture plants had the peak of sheath length at a higher rank (7) compared with wide and narrow intercrop (both 6). Beyond rank 6, monoculture plants had the largest sheath lengths (significant for all ranks).

**Fig. 4.**
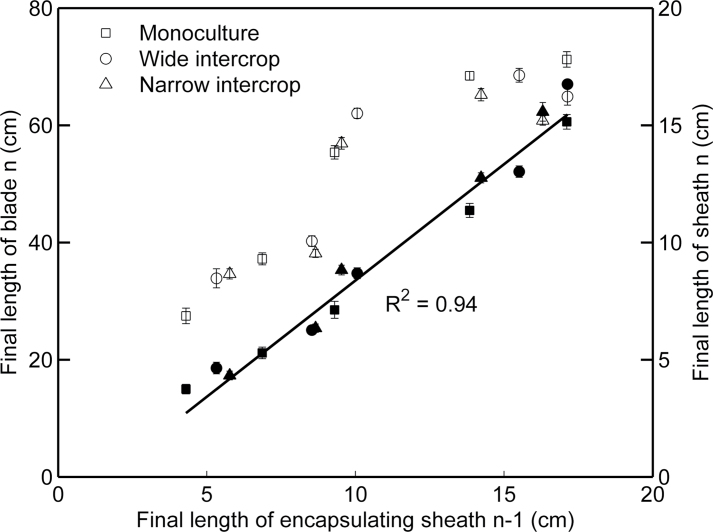
Final length of the blade (filled symbols, primary *y*-axis) and sheath (open symbols, secondary *y*-axis) of phytomers 3–7 plotted against the final length of the encapsulating sheath (i.e. the sheath of the preceding phytomer) in monoculture (squares, *n*=11), wide intercrop (circles, *n*=7), and narrow intercrop (triangles, *n*=7). Error bars indicate the SE. Monoculture plants had the peak of sheath length at rank 7, and both wide and narrow intercrop had the peak at rank 6.

The relationship between final blade length and the length of the encapsulating sheath (i.e. the sheath of the previous phytomer, which represents the length of the whorl that a blade grew through before maturity) was linear and independent of the treatment for ranks 3–7 (Fig. 4). Data for rank 1 and 2 were missing. No stable relationships were found between blade and sheath lengths beyond rank 7. Final sheath length also increased with the length of the encapsulating sheath, but the relationship levelled off for lengths of the encapsulating sheaths >13cm.

### Blade elongation duration and rate

**Fig. 5.**
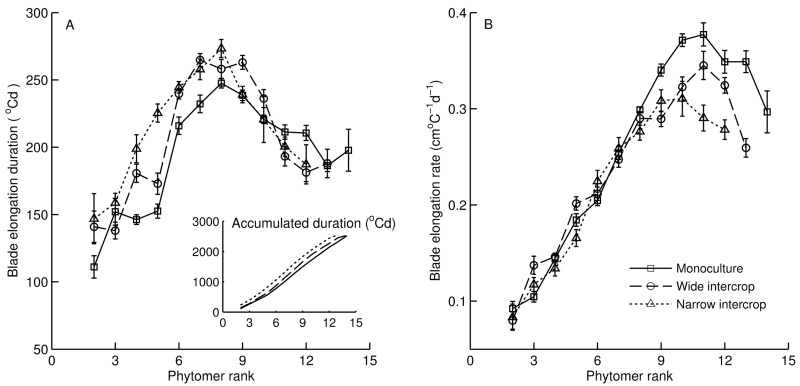
(A) Blade elongation duration and (B) blade elongation rate in monoculture (squares, *n*=8), wide intercrop (circles, *n*=9), and narrow intercrop (triangles, *n*=7) versus phytomer rank. Inset (A): accumulated elongation duration from phytomer 2 to the last. Error bars indicate the SE.

The duration of the visible blade elongation from blade tip appearance to collar emergence, plotted against phytomer rank, showed a bell-shaped curve in all treatments (Fig. 5A). Up to the peak, monoculture had the shortest blade elongation duration and lowest accumulated duration (inset in Fig. 5A). Beyond the peak, the trend reversed: monoculture gradually showed the longest elongation duration. A similar shape was found for average blade elongation rate, with the peak at rank 10 or 11 (Fig. 5B). In contrast, no significant differences were found in blade elongation rate as a function of rank among treatments below rank 10. For higher ranks, the comparison was confounded with the difference in final leaf number.

### Red:far-red ratio and photosynthetically active radiation

**Fig. 6.**
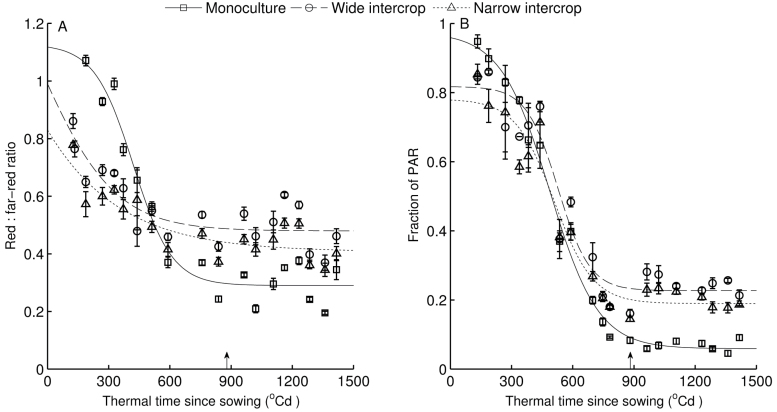
(A) Red:far-red ratio and (B) fraction of PAR at the soil level as a function of thermal time since sowing in monoculture, wide intercrop, and narrow intercrop. R:FR values represent averages of four values (sensor facing north, east, south, and west) and PAR values represent averages of four or five values measured in one plot. Arrows indicate wheat harvest time (887 °Cd). Error bars indicate the SE.

In the early stages of canopy development (before~500 °Cd) the highest R:FR and fractions of PAR reaching the soil surface were found in monoculture canopies, while narrow intercrop canopies showed the lowest values (Fig. 6). However, both R:FR and PAR fractions decreased faster in monoculture than in intercrop canopies, resulting in monoculture canopies having the lowest R:FR and PAR fractions in all treatments. In the end, R:FR stabilized at ~0.29, 0.48, and 0.41, and PAR fraction at ~0.06, 0.23, and 0.19 (Supplementary Table S1 at *JXB* online) in monoculture, wide intercrop, and narrow intercrop, respectively. The values in wide intercrop canopies were always above those in narrow intercrop canopies.

## Discussion

The aim of this study was to assess the developmental response of maize to the growth conditions in mixed cultivation with wheat. In the intercrop treatments used here, maize seedlings experienced strong competition for light by neighbouring wheat plants, which were ~50cm tall at maize emergence (Fig. 1). Mixed cultivation lowered the early R:FR in maize due to reflection of low R:FR light by wheat, and PAR was initially lower in intercrops due to shading by adjacent wheat plants (Fig. 6). The real extent of R:FR and PAR reduction over the whole day must probably have been greater than presented here, since the measurements were made around noon, when shading is at the lowest point of the day. Maize development in intercrops was significantly affected, right from the start, as shown by decelerated leaf appearance and collar emergence rates, and enhanced final blade and sheath lengths of low ranks. A close relationship between blade and sheath length at low ranks was found across treatments (Fig. 4), explaining why intercropped plants have longer blades. Substantial differences among treatments were found in blade elongation duration in low ranks, but the blade elongation rate was not affected (Fig. 5).

Based on these quantitative findings and previously established rules in coordination of maize development (Skinner and Nelson, 1994, 1995; Fournier and Andrieu, 2000*a*; Fournier *et al.*, 2005; Andrieu *et al.*, 2006; Verdenal *et al.*, 2008), it is inferred that plasticity in leaf appearance and final length of blades and sheaths emerge as a result of coordination of developmental processes. Early modification of the light environment of maize seedlings by wheat plants in an intercrop generates local responses at the phytomer level which subsequently interact, apparently according to generic rules, to shape development of maize whole-plant architecture during the remainder of the season.

### Enhanced sheath length during early development is a shade avoidance response

The crossover in R:FRs and fraction of PAR at soil level across treatments occurred at ~500 °Cd (Fig. 6). This is when intercrops had 5–6 and monoculture had seven fully expanded leaves (Fig. 2). Final sheath length is reached soon after collar emergence (Lafarge *et al.*, 1998; Fournier and Andrieu, 2000*a*). The crossover in sheath length across treatments occurred around phytomer 6 (Fig. 3D), and that in blade length/width ratio occurred around phytomer 8 (Fig. 3C). Hence, it seems that some of the changes in treatment effects on organ size occurred around the same time as the crossover in R:FRs and fraction of PAR at the soil level, suggesting a relationship between organ size and radiation conditions. Low R:FR or low blue light intensity enhance sheath extension in grasses (Casal *et al.*, 1985), which allows plants to avoid future shading by neighbours (Corré, 1984; Ballaré, 1999). Moreover, longer sheaths were found at high population density compared with regular population density in maize (Andrieu *et al.*, 2006), which was attributed to neighbour-induced early drops in R:FR. This leads to the inference that enhanced sheath length of low ranks (Fig. 3D) was associated with a reduction in R:FR and PAR fraction at the soil surface, which are intimately related (Evers *et al.*, 2006). The increase in sheath length in subsequent early phytomers supports the idea that collar emergence triggers the decline of sheath elongation rate (Fournier and Andrieu, 2000*a*), and is responsible for propagating differences in length created on early phytomers because of the linear relationship between the length of successive sheath ranks and between blade length and sheath length of the preceding phytomer (Fig. 4) (Andrieu *et al.*, 2006).

### Leaf appearance and final leaf number are affected by sheath length at low ranks and by feedback between leaf emergence and initiation

Leaf appearance was significantly delayed in the two intercrop treatments. For example, in comparison with monoculture maize, the appearance of leaf 10 was delayed by 67.7 °Cd in the wide intercrop and by 157.8 °Cd in the narrow intercrop. The extent of leaf appearance delay caused by intercropping was much larger than the effect of high population density in maize compared with normal density (24.2 °Cd for leaf 10) (Andrieu *et al.*, 2006). Likewise, Page *et al.* (2011) found that an artificially low R:FR resulted in 1.1 fewer leaf tips at the 10-leaf tip stage of maize. Since temperature did not materially differ between treatments and leaf appearance of maize is comparatively insensitive to N supply within a wide range (Radin, 1983; Vos *et al.*, 2005), it appears plausible that early changes in light environment cause the delay in leaf appearance.

Padilla and Otegui (2005) found that there is a positive linear relationship between the number of appeared and initiated leaves in maize and that this relationship is conservative over varieties and environmental conditions. The present observation of larger phyllochron in intercropped maize as compared with monoculture maize thus indicates that plastochron is greater in intercropped maize than in monoculture maize. There was only a small effect on tassel initiation time, an event that is mainly determined by temperature and photoperiod (Muchow and Carberry, 1989; Birch *et al.*, 1998*b*). As a consequence, the final number of leaves was lower in intercropping, which supports the positive association derived by Sadras and Villalobos (1993) that final leaf number is equal to the product of thermal time duration from emergence to tassel initiation and rate of leaf primordium initiation plus number of leaf primordia in the embryo. The comparable ratios of leaf initiation rate and leaf appearance rate between monoculture and intercrops indicate that the change in leaf initiation rate and leaf appearance rate is consistent, which supports the hypothesis that leaf initiation is coordinated with leaf emergence (Padilla and Otegui, 2005).

There are two possible mechanisms for the coordination between leaf initiation and leaf emergence. First, apex growth and primordia initiation depend on carbon supply of photosynthetic leaves, especially the first leaf, and shade reduces this carbon supply (Felippe and Dale, 1973). Secondly, apex development is influenced by signals transferred from the emerging leaf (Bernier, 1988) or from the roots (Pons *et al.*, 2001; Wasternack, 2007). Signalling has been shown to play a role in the floral transition of maize: a transmissible signal in the leaf elicits the transformation of the shoot apex to reproductive development (Colasanti *et al.*, 1998). Thus it is inferred that there is a positive feedback between leaf emergence and leaf initiation in which a delay in leaf emergence would be amplified by the delay in leaf initiation, and vice versa. This would explain why in the present experiment leaf appearance continued to diverge between treatments.

It is concluded that the effect of the intercrop treatments on leaf appearance can be attributed to two factors: length of the sheath whorl and leaf initiation rate at the stem apex. For a given rate of leaf elongation, leaf emergence occurs later when the preceding sheaths are longer. Therefore, an increase in sheath length due to shade avoidance could postpone the time of leaf emergence, and this is a likely contributing factor to delayed initiation of new leaves.

### Final blade length distribution along the stem is driven by sheath length and tassel initiation

**Fig. 7.**
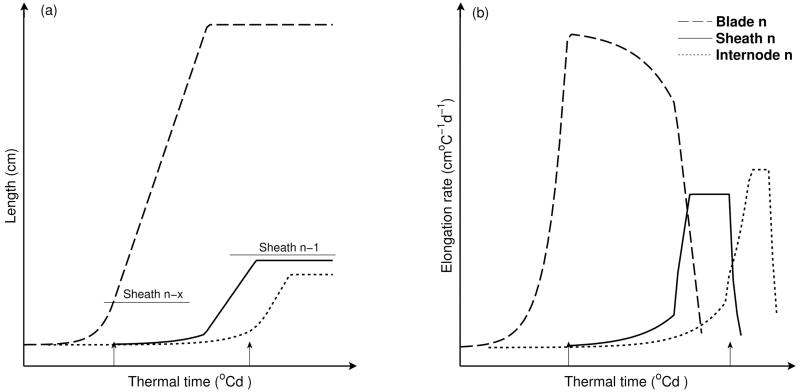
Model of the lengths (A) and elongation rate (B) of the blade, sheath, and internode of a phytomer against thermal time. Arrows represent tip emergence (left arrow) and collar emergence (right arrow). Horizontal lines in (A) represent the length of the sheath with its collar at the highest position of the plant, at tip emergence (left line) and collar emergence (right line). In this model, the rate of leaf initiation is influenced by tip emergence. The blade follows quasi-exponential growth until emergence of the tip. The associated internode is initiated about half a plastochron after the blade is initiated, and then follows exponential growth until collar emergence (Fournier and Andrieu, 2000*a*). Before tassel initiation, tip emergence triggers sheath initiation. After tassel initiation, sheaths are initiated according to a repetitive scheme (Andrieu *et al.*, 2006). The growth of the sheath gradually reduces the growth rate of the associated blade. Collar emergence triggers the growth shift between sheath and internode (Fournier and Andrieu, 2000*a*) and consequently inhibits sheath length increase.

A likely cause of the enhanced duration of blade elongation at low ranks in the intercropping treatments (Fig. 5A) was the link between elongation duration and sheath length. Due to the coordination between tip emergence and sheath initiation (Andrieu *et al.*, 2006; Parent *et al.*, 2009), longer sheaths delayed tip emergence and therefore increased the duration of blade elongation (Verdenal *et al.*, 2008). In addition, extension of the sheath progressively reduces the growth rate of the blade since they share the same growing zone (Fig. 7B) (Schnyder *et al.*, 1990; Skinner and Nelson, 1994). After tassel initiation, sheaths initiated faster, independent of leaf tip emergence. This is supported by the observation that the collar emergence diverged before rank 8, while afterwards collars emerged at similar rates across treatments (Fig. 2). This explained the plateau in leaf length for the middle phytomers. The reduction of final blade length in high ranks was probably due to a reduction in relative blade elongation rate and undelayed sheath initiation and fast extension (Andrieu *et al.*, 2006). This provides a mechanistic explanation for how the typical bell shape of the final blade length distribution along the stem was formed (Fig. 3A), which has been found in many studies on maize (Fournier and Andrieu, 1998; Ma *et al.*, 2007) and is generally found in cereals, such as wheat (Evers *et al.*, 2005), sorghum (*Sorghum bicolor*) (Lafarge *et al.*, 2002), barley (*Hordeum vulgare*) (Buck-Sorlin *et al.*, 2005), and rice (Tivet *et al.*, 2001).

### Towards a general conceptual model of maize shoot development in response to early competition

From the present results, a conceptual model of maize shoot development can be derived that captures the effects of strong competition during early development, such as in wheat–maize intercropping (Fig. 7). Early low R:FR and PAR in the intercrop enhance the sheath length of the lower phytomers (including coleoptile) and decelerate leaf initiation. This then slows the emergence of leaf tips and collars, which is propagated to later formed phytomers by the feedback between leaf emergence and initiation. The time of switch to the reproductive phase was similar across treatments. Thus, because of the lower leaf initiation rate in intercrop, the final leaf number was lower in the intercropped maize than in the monoculture. These concepts of shoot development underlie plasticity in leaf emergence and organ size in response to environmental cues for competition, and can be scaled up to a whole-plant response.

### Relationship between maize developmental response, light interception, and yield

Crop production is closely related to the cumulative intercepted radiation (Monteith and Moss, 1977; Zhang *et al.*, 2008). Maize and wheat grown in relay-intercrop, as in this study, where the growing seasons of the two species overlap only partly in time, have the ability to intercept more light over the season than either of the single crops would be able to do. For this to be realized, plant adaptation might be required to fill the gaps that are left in the sowing pattern for later sowing of maize. Zhang and Li (2003) reported strong overyielding in border rows of wheat in wheat–maize intercrop. Maize seedlings are competitively weaker than wheat, which was already 50cm tall at the maize seedling stage. As shown here, maize adapts by shade avoidance, which might have acted to mitigate the yield losses that could possibly have occurred, due to shading by wheat, if shade avoidance had not taken place. A simulation model that takes into account the structural adaptations and calculates light interception at the organ level (Vos *et al.*, 2010) would be helpful in evaluating the value of these adaptations in enhancing light interception and carbon assimilation. Understanding such responses can help identify intercrop designs and plant genotypes that maximize light interception and yield in a mixed stand. Whilst such above-ground responses are undoubtedly of key importance for the functioning and productivity of the crop, it should also be considered that below-ground processes could equally affect resource capture and productivity (Li *et al.*, 2007). Hence, this research has only been a first step to link intercrop productivity to crop responses at the plant and phytomer level, and the inter-phytomer regulation of plant development. In intercropping studies there needs to be special interest also in the responses of roots (de Kroon, 2007) and in coupling above- and below-ground plant development and architecture. It is believed that further work in this domain is important and promises to contribute eventually to efficient land use, high crop productivity, and food security.

## Supplementary data

Supplementary data are available at *JXB* online.

Figure S1. Calculation of weighting factor of different PAR measurement positions in intercropping.

Figure S1. Schematic diagrams of the experimental layout.

Figure S2. Example of using the beta function to derive blade elongation duration.

Table S1. Fitting parameters for the R:FR ratio and PAR dynamics in three treatments.

Table S2. Coordination between leaf initiation and leaf appearance.

Supplementary Data
