# Supplementary material for: Early competition shapes maize whole-plant development in mixed stands
Source: J Exp Bot. 2013 Dec 4;65(2):641–53. doi: 10.1093/jxb/ert408 (PMC3904716; doi:10.1093/jxb/ert408)
Supplement: Supplementary Data [file supp_65_2_641__index.html]

Early competition shapes maize whole-plant development in mixed stands — Early competition shapes maize whole-plant development in mixed stands — Supplementary Data 

# Early competition shapes maize whole-plant development in mixed stands

## Supplementary Data

Data files

**Files in this Data Supplement:**

- Supplementary Data - Supplementary Data
