# Supplementary material for: Early competition shapes maize whole-plant development in mixed stands
Source: J Exp Bot. 2013 Dec 4;65(2):641–53. doi: 10.1093/jxb/ert408 (PMC3904716; doi:10.1093/jxb/ert408)
Supplement: Supplementary Data [file supp_ert408_jexbot106666_file001.pdf]

Supporting information

Early competition shapes maize whole plant development in mixed stands

Junqi Zhu, Jan Vos, Wopke van der Werf, Peter E. L. van der Putten, Jochem B. Evers

Method S1

Calculation of weighting factors of different PAR measurement positions in intercropping. Because the distance between different measurement locations was not equal (see PAR measurement position one to five in Fig.1 counting from left to right), we calculated the representative length of each measurement and its weight relative to the width of maize strip based on several assumptions.

Before wheat harvest (taking wide intercrop as an example):

a) Assume the space occupied by the wheat border row is 12.5cm (same for narrow intercrop) and the remaining 31.5 cm (12.5 cm for narrow intercrop) between wheat and maize row is occupied by maize, so the width of maize strip is 75 + 31.5*2 = 138 cm (100 cm for narrow intercrop)

b) The mean PAR values of positions one and two represent the 31.5 cm (12.5 cm) between wheat and maize row; same for positions four and five. Mean values of position two and three represent the space from the maize row to the middle of two maize rows (37.5 cm), same for mean of position three and four.

c) Subsequently, the weighting factors based on the representative length and maize strip width are 0.114, 0.250, 0.272, 0.250, 0.114 (wide intercrop) and 0.063, 0.250, 0.375, 0.250, 0.063 (narrow intercrop) before wheat harvest for position one to five.

After wheat harvest:

The width of maize strip is 150 cm (width in monoculture maize) and mean value of position one and two represent 37.5cm. Therefore, the weighting factors are 0.125, 0.25, 0.25, 0.25, 0.125 for positions one to five for wide intercrop and narrow intercrop, respectively,

Fig. S1

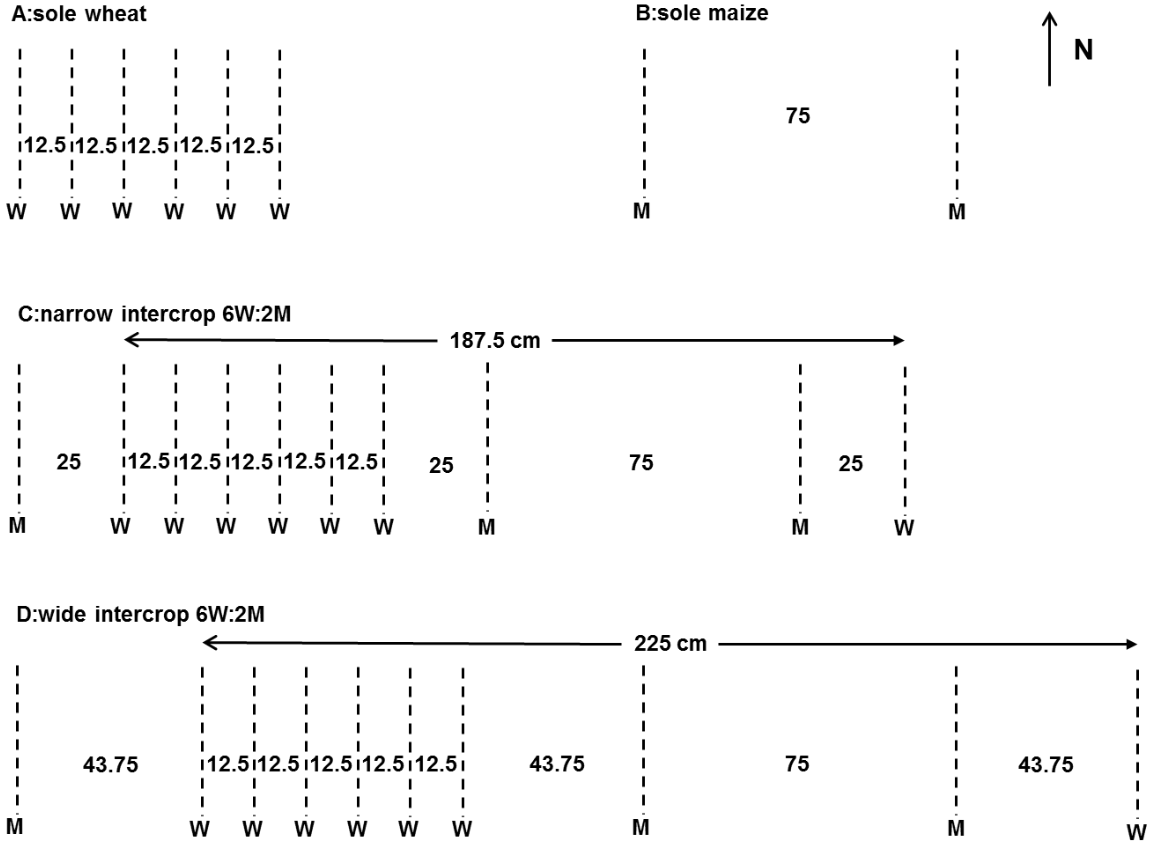

Fig. S1: Layout of wheat-maize intercrop and monocultures in four treatments (A) sole wheat, row distance 12.5 cm (B) sole maize, row distance 75 cm (C) narrow intercrop according to a non-replacement design, i.e. the sum of relative densities is 1.2; (D) wide intercrop according to a replacement design, i.e. the sum of the relative densities of maize and wheat (each as compared to the density in monoculture) is 1. In this case, the distance between adjacent maize and wheat equals the average of the row distances in monoculture wheat and monoculture maize. In (C), this distance is smaller than the average row distance in sole crops. The plot size is 6 by 6 meters, with some bare space for narrow intercrop. For both two intercrops, they have 3 wheat strips and 2 maize strips between them. And there is also one row of maize beside the wheat strip at each side.

Fig. S2

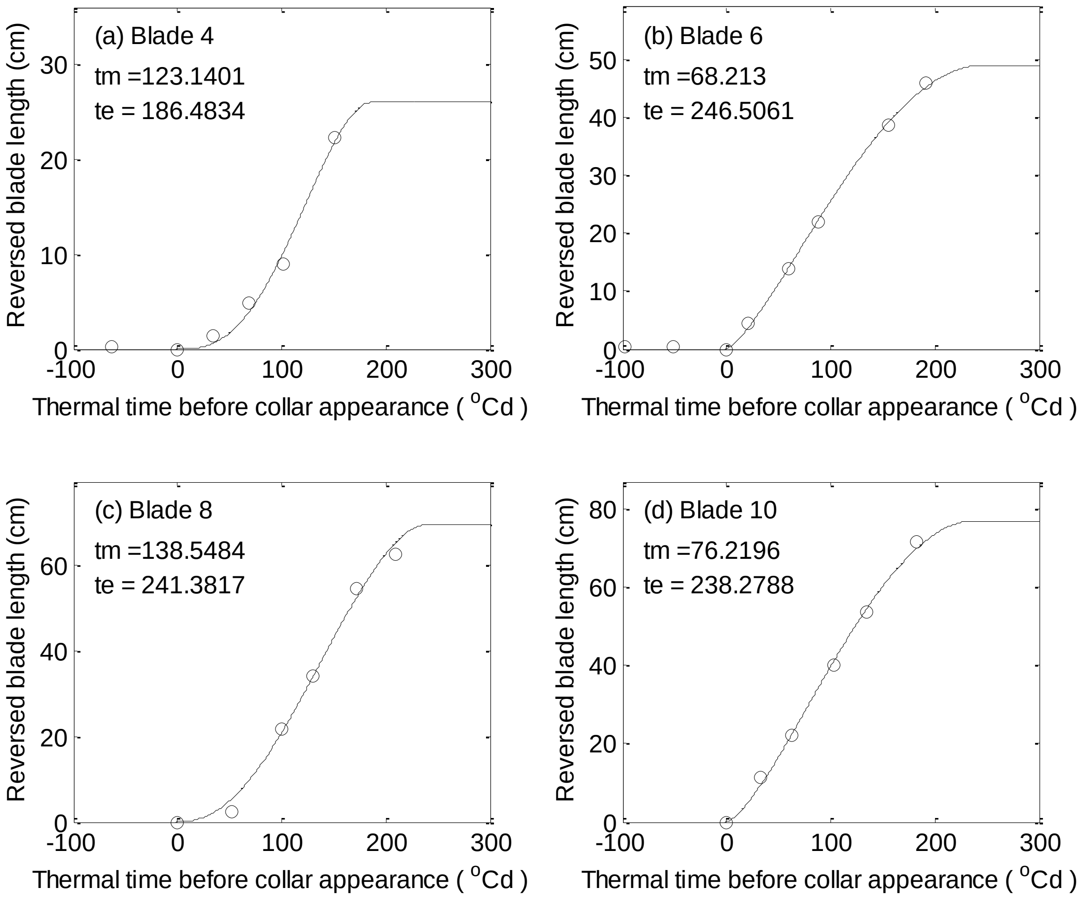

Fig. S1: Example of using beta function to derive blade elongation duration for each individual blade (Eq.1)

a) Collar emergence time was set to zero °Cd, because collar emergence was estimated more reliably than tip appearance by the blade dynamics data.
b) Subsequently, the curve was reversed by taking final blade length minus measured length versus absolute thermal time.
c) Blade elongation duration t_e was fitted by the beta function (Eq. 1 in the main text).

Table S1

Fitting parameters for R:FR and PAR dynamic in three planting systems

		ζ_{min}	ζ_{max}	k (°Cd^{-1})	t_{i} (°Cd)
	Monoculture	0.29	1.13	$-1.1 \cdot 10^{-2}$	420
R:FR	Wide intercrop	0.48	1.63	$-5.4 \cdot 10^{-3}$	-43
	Narrow intercrop	0.41	8.68	$-3.0 \cdot 10^{-3}$	-981
	Monoculture	$5.9 \cdot 10^{-2}$	0.97	$-8.4 \cdot 10^{-3}$	486
PAR	Wide intercrop	0.23	0.82	$-1.3 \cdot 10^{-3}$	528
	Narrow intercrop	0.19	0.78	$-1.1 \cdot 10^{-3}$	509

Where ζ is the red : far-red ratio. ζ_{min} and ζ_{max} are the lower and upper asymptotes (dimensionless), k is the slope at the inflection point (°Cd^{-1}), and t_{i} is the thermal time of the inflection point (°Cd).

Table S2

Coordination between leaf initiation and leaf appearance

Initiated leaves (#)	8	9	10	11	12	13	14	15
Appeared leaves (#)	3	3.63	4.26	4.89	5.52	6.15	6.78	7.41

Estimation equation is: Appeared leaves = 0.63 * (Initiated leaves – 8) + 3. Initial estimation point was set to 3 appeared leaves and 8 initiated leaves, subsequently 0.63 leaves appeared per initiated leaf (Padilla and Otegui, 2005).
